# Association between plasma levels of homocysteine, folate, and vitamin B_12_, and dietary folate intake and hypertension in a cross-sectional study

**DOI:** 10.1038/s41598-020-75267-3

**Published:** 2020-10-28

**Authors:** Takashi Tamura, Nagato Kuriyama, Teruhide Koyama, Etsuko Ozaki, Daisuke Matsui, Yuka Kadomatsu, Mineko Tsukamoto, Yoko Kubo, Rieko Okada, Asahi Hishida, Tae Sasakabe, Sayo Kawai, Mariko Naito, Naoyuki Takashima, Aya Kadota, Keitaro Tanaka, Megumi Hara, Sadao Suzuki, Hiroko Nakagawa-Senda, Toshiro Takezaki, Ippei Shimoshikiryo, Hiroaki Ikezaki, Masayuki Murata, Isao Oze, Hidemi Ito, Haruo Mikami, Yohko Nakamura, Kiyonori Kuriki, Kokichi Arisawa, Hirokazu Uemura, Kenji Takeuchi, Kenji Wakai

**Affiliations:** 1 Department of Preventive Medicine, Nagoya University Graduate School of Medicine, 65 Tsurumai-cho, Showa-ku, Nagoya, 466-8550 Japan; 2 Department of Epidemiology for Community Health and Medicine, Kyoto Prefectural University of Medicine, Kyoto, Japan; 3 Department of Public Health, Aichi Medical University School of Medicine, Nagakute, Japan; 4 Department of Oral Epidemiology, Hiroshima University Graduate School of Biomedical and Health Sciences, Hiroshima, Japan; 5 Department of Public Health, Shiga University of Medical Science, Otsu, Japan; 6 Department of Public Health, Faculty of Medicine, Kindai University, Osakasayama, Japan; 7 Department of Preventive Medicine, Faculty of Medicine, Saga University, Saga, Japan; 8 Department of Public Health, Nagoya City University Graduate School of Medical Sciences, Nagoya, Japan; 9 Department of International Island and Community Medicine, Kagoshima University Graduate School of Medical and Dental Sciences, Kagoshima, Japan; 10 Department of Environmental Medicine and Infectious Disease, Kyushu University Graduate School of Medical Sciences, Fukuoka, Japan; 11 Division of Cancer Epidemiology and Prevention, Aichi Cancer Center Research Institute, Nagoya, Japan; 12 Division of Cancer Information and Control, Aichi Cancer Center Research Institute, Nagoya, Japan; 13 Division of Descriptive Cancer Epidemiology, Nagoya University Graduate School of Medicine, Nagoya, Japan; 14 Cancer Prevention Center, Chiba Cancer Center Research Institute, Chiba, Japan; 15 Laboratory of Public Health, Division of Nutritional Sciences, School of Food and Nutritional Sciences, University of Shizuoka, Shizuoka, Japan; 16 Department of Preventive Medicine, Institute of Biomedical Sciences, Tokushima University Graduate School, Tokushima, Japan; 17 College of Nursing Art and Science, University of Hyogo, Akashi, Japan

**Keywords:** Metabolic disorders, Epidemiology, Preventive medicine

## Abstract

There are few studies examining the association between homocysteine (Hcy) level and the risk of hypertension with consideration for folate and vitamin B_12_ as related to Hcy level. We simultaneously examined the associations of plasma levels of Hcy, folate, and vitamin B_12_, and dietary folate intake with the prevalence of hypertension. Participants included 1046 men and 1033 women (mean age ± standard deviation: 56.0 ± 8.9 years) in the Japan Multi-Institutional Collaborative Cohort Study. Dietary folate intake was estimated using a validated food frequency questionnaire. Hypertension was defined based on measured blood pressure and use of antihypertensive medication. A total of 734 participants (35.3%) had hypertension. Multivariate-adjusted odds ratios of hypertension for the highest quartile group of Hcy were 2.36 (95% CI 1.41–3.96) in men and 1.86 (95% CI 1.11–3.11) in women, as compared with the lowest group (*P* for trend = 0.014 and 0.005, respectively). Dietary folate intake was not correlated with hypertension in both men and women (*P* for trend = 0.099 and 0.703, respectively). Plasma vitamin B_12_ was positively associated with hypertension only in women (*P* for trend = 0.027). Plasma Hcy level was positively linked with hypertension after controlling for covariates, including folate and vitamin B_12_.

## Introduction

Hypertension is a major public health concern worldwide^1^, which is influenced by both genetic and environmental factors^2^. Epidemiological studies have revealed that multiple environmental factors are associated with hypertension, such as obesity, alcohol consumption, sodium intake, and physical inactivity^3–6^. A better understanding of modifiable risk factors for hypertension is useful for early detection and prevention of hypertension, which contributes to reducing this disease and its associated complications.

Homocysteine (Hcy) is a non-proteinogenic α-amino acid produced by the demethylation of methionine. Hyperhomocysteinemia is known as an established risk factor for cardiovascular disease (CVD) and stroke^7,8^. To date, attention has been focused on the fact that this adverse effect caused by hyperhomocysteinemia might be partially mediated by a positive association between Hcy levels and blood pressure^9^. Increased plasma Hcy causes oxidative stress and endothelial dysfunction, which leads to vascular constriction, stiffness, and decrease in the vasodilation by nitric oxide, resulting in an increase in blood pressure^9–11^. Some previous observational studies have demonstrated positive associations between Hcy levels and blood pressure or the risk of hypertension^12–15^. However, other studies including large prospective cohort studies do not support these associations^16–21^. Thus, the association between Hcy levels and risk of hypertension remains inconclusive. A recent meta-analysis of relevant randomized trials also revealed that folate supplementation is effective in reducing blood pressure and Hcy levels among patients with hypertension and hyperhomocysteinemia^22^. In addition, observational studies showed that dietary folate intake and folate level in blood were inversely associated with hypertension^23–25^. These findings suggest that it is important to consider dietary intake and plasma levels of folate typically involved in the metabolism of Hcy in relation to the association between Hcy levels and risk of hypertension. Interestingly, a previous study suggested that vitamin B_12_ as related to Hcy metabolism is also inversely associated with blood pressure^26^. Nevertheless, few studies have examined the association with these nutrients. It is also of interest to examine the hypothesis that folate and vitamin B_12_ are independently associated with the risk of hypertension in a general population. The evidence for this hypothesis is weak, although these vitamins are essential in the metabolism of methionine and are major nutritional determinants of plasma Hcy levels^27^. Furthermore, the association of Hcy with hypertension may be dependent on folate and/or vitamin B_12_. Therefore, to consider these nutrients involved in Hcy metabolism in addition to Hcy itself should be important.

Thus, in the present study, we aimed to simultaneously examine the association of plasma levels of Hcy, folate, and vitamin B_12_, and dietary folate intake with the prevalence of hypertension, after controlling for multiple confounding factors, using a relatively large sample size in a cross-sectional study conducted among a general Japanese population.

## Methods

### Participants

Participants in the present study were enrolled in the Japan Multi-Institutional Collaborative Cohort (J-MICC) Study^28^. The J-MICC Study is a large cohort study in Japan, launched in 2005 to identify interactions between genetic and lifestyle factors for lifestyle-related diseases, including any cancer. The details and rationale of the J-MICC Study have been described elsewhere^29^. Briefly, participants completed a self-administered questionnaire on lifestyle and medical status, and then donated a peripheral blood sample during the baseline survey. The J-MICC Study enrolled residents in the community, health check examinees, and patients at a cancer hospital. All participants in the study provided their written informed consent. The study protocol was approved by the ethics committees of Nagoya University Graduate School of Medicine, Aichi Cancer Center, Chiba Cancer Center, Nagoya City University Graduate School of Medical Sciences, Shiga University of Medical Science, Kyoto Prefectural University of Medicine, Kyushu University Graduate School of Medical Sciences, Saga University Faculty of Medicine, Kagoshima University Graduate School of Medical and Dental Sciences, Tokushima University Graduate School, and University of Shizuoka participating in the J-MICC Study. The present study was conducted according to the principles expressed in the World Medical Association Declaration of Helsinki.

A total of 92,631 participants were recruited from 14 different areas throughout Japan between 2004 and 2014 (the dataset used in the present study was fixed on February 15, 2019). Of the total population, 2845 participants in eight study areas underwent measurement of plasma concentrations of Hcy, folate, and vitamin B_12_. Among the total, we excluded all participants from two areas (n = 763) because of no available data for blood pressure. We further excluded those with missing data for blood pressure and use of antihypertensive medication (n = 1 and 2, respectively). Thus, the present study finally included 2079 individuals (1046 men and 1033 women) from six study areas (Okazaki, Shizuoka, Takashima, Kyoto, Saga, and Kagoshima) with complete data for the analysis.

### Assessment of lifestyle factors, dietary folate intake, and blood pressure

We used a self-administered questionnaire that included the following demographic characteristics and lifestyle and medical factors: alcohol consumption, smoking status, education level, physical activity, self-reported psychological stress, sleeping hours, family and personal medical history, current medication, and menstruation in women.

Physical activity was estimated as metabolic equivalent hours per day, based on the frequency and duration of daily and leisure time activities^30,31^. Ethanol intake (g/day) was estimated for current drinkers (defined as those who consumed alcohol at least once a week during the last year), based on the reported consumption frequency and amount consumed per one time for six alcoholic beverages (Japanese sake, shochu, shochu-based cocktails, beer, whisky, and wine)^32^. Dietary folate and energy intakes were estimated using a validated short food frequency questionnaire^33–36^; Spearman correlation coefficients between estimated intakes according to the questionnaire and 3-day weighed dietary records were 0.41 in men and 0.36 in women for folate, and 0.36 in men and 0.37 in women for total energy. Dietary folate intake per day was adjusted for total energy intake using the nutrient density method^37^, which computes the dietary folate intake per 1000 kcal of daily total energy intake. We did not consider the loss of dietary folate intake due to the cooking with heat for foods because of limited data. In addition, this study did not include participants who reported folate supplement use, and thus we did not consider folate supplementation in examining the associations.

Height and weight were measured directly on the day of the survey; body mass index (BMI) was calculated as weight in kilograms divided by the square of height in meters (kg/m^2^). Each participant’s blood pressure was measured by a nurse or trained staff during health checks, using an automated blood pressure measurement monitor, with the patient in a seated position^38^.

### Blood samples and measurements of plasma Hcy, folate, and vitamin B_12_

Fasting peripheral blood was drawn from participants at health checks, which was collected in a 7-mL EDTA-2Na-containing vacuum tube for plasma. Plasma separated from whole blood by using a centrifuge was stored at approximately − 80 °C until analysis. Plasma concentration of Hcy was determined using high-performance liquid chromatography. Concentrations of folate and vitamin B_12_ were determined using chemiluminescent enzyme immunoassay. All measurements were conducted at SRL Co., Ltd., Hachioji, Japan.

### Statistical analyses

Participant characteristics were presented as mean ± standard deviation (SD) for continuous variables and as number and proportion for categorical variables, according to quartiles of plasma Hcy level by sex. Differences in the mean or proportion between quartiles of Hcy level were tested using analysis of variance or the chi-squared test, respectively. Difference in the mean of Hcy level between those with hypertension and those without was tested using the Mann–Whitney *U* test. The Spearman correlation coefficient was calculated between plasma folate levels and energy-adjusted dietary folate intake. We performed all analyses stratified by sex, as Hcy levels differ greatly by sex^39^.

Hypertension was defined according to any of the following criteria: systolic blood pressure ≥ 140 mmHg, diastolic blood pressure ≥ 90 mmHg, or use of antihypertensive medication^40,41^. Crude, age-adjusted, and multivariate-adjusted odds ratios (ORs) and 95% confidence intervals (CIs) for hypertension were estimated according to quartile levels of plasma Hcy, folate, and vitamin B_12_, and dietary folate intake using unconditional logistic regression models. The multivariate-adjusted model included the following covariates: age (as a continuous variable), alcohol consumption (never, former, current drinker who consumed < 23 g/day ethanol, current drinker who consumed ≥ 23 g/day ethanol), smoking status (never, former, current), education level (9, 10–15, ≥ 16 years), BMI (< 18.5, 18.5 to < 25.0, ≥ 25.0 kg/m^2^), physical activity (as a continuous variable), psychological stress (not at all, not much, a little, a lot), sleeping hours (< 6, 6 to < 8, ≥ 8 h), family history of parental hypertension (yes, no for both father and mother), medical history of diabetes mellitus (yes, no), medical history of dyslipidemia (yes, no), medical history of CVD (yes, no), medical history of stroke (yes, no), menstruation for women (premenopausal, perimenopausal, postmenopausal), and study area (Okazaki, Shizuoka, Takashima, Kyoto, Saga, Kagoshima). In an additional multivariate-adjusted model, we further controlled for plasma concentrations of Hcy, folate, and vitamin B_12_, and dietary folate intake each other (as a continuous variable, respectively). Participants with missing data for covariates were included as additional categories in the analysis. The linear trend for risk was evaluated using a continuous variable for plasma levels of Hcy, folate, and vitamin B_12_, and dietary folate intake, respectively.

A two-tailed *P* value of < 0.05 was considered statistically significant. All statistical analyses were performed using SAS 9.4M5, which runs on SAS University Edition (SAS Institute Inc., Cary, NC, USA).

## Results

Baseline characteristics of 2079 study participants (1046 men and 1033 women) according to the quartiles of plasma Hcy level by sex are shown in Table 1. Mean age ± SD was 56.0 ± 8.9 years, and the proportion of men was 50.3%. Current drinkers accounted for 79.6% of men and 34.8% of women. There was no difference in the mean of age among quartiles of Hcy level. Plasma Hcy concentration was inversely associated with plasma levels of folate and vitamin B_12_, dietary folate intake (in men), and energy intake. Spearman correlation coefficients between plasma folate level and energy-adjusted dietary folate intake were 0.105 in men and 0.129 in women (*P* < 0.001, respectively). The distributions of alcohol consumption, education level, BMI, physical activity, psychological stress, sleeping hours, and family history of parental hypertension did not significantly differ among quartiles of Hcy level. Men with higher plasma Hcy levels were more likely to be current smokers; this association was not observed in women. There were no differences in the distribution of medical histories of diabetes mellitus, dyslipidemia, and CVD among quartiles of Hcy level, and that of stroke differed only in women. Differences in the distribution of study areas were observed among quartiles of Hcy level.

**Table 1:** Participant characteristics according to quartiles (Q1–Q4) of plasma Hcy level, by sex.

Characteristics	Men (n = 1046)					Women (n = 1033)				
	Q1	Q2	Q3	Q4	*P* value	Q1	Q2	Q3	Q4	*P* value
**No. of participants**	227	275	266	278		223	264	266	280	
**Age (years ± SD)**	56.2 ± 8.9	56.5 ± 8.6	56.0 ± 8.9	54.9 ± 9.8	0.177	55.5 ± 8.9	55.5 ± 9.0	56.6 ± 8.2	56.9 ± 8.7	0.131
**Alcohol consumption (n, %)**										
Never	41 (18.1)	39 (14.2)	47 (17.7)	65 (23.4)	0.177	137 (61.4)	159 (60.2)	175 (65.8)	191 (68.2)	0.164
Former	3 (1.3)	5 (1.8)	3 (1.1)	9 (3.2)		2 (0.9)	5 (1.9)	1 (0.4)	4 (1.4)	
Current, < 23 g/day ethanol	83 (36.6)	108 (39.3)	109 (41.0)	98 (35.3)		76 (34.1)	94 (35.6)	79 (29.7)	71 (25.4)	
Current, ≥ 23 g/day ethanol	100 (44.1)	122 (44.4)	107 (40.2)	106 (38.1)		8 (3.6)	6 (2.3)	11 (4.1)	14 (5.0)	
Unknown	0 (0.0)	1 (0.4)	0 (0.0)	0 (0.0)		0 (0.0)	0 (0.0)	0 (0.0)	0 (0.0)	
**Smoking status (n, %)**										
Never	66 (29.1)	76 (27.6)	68 (25.6)	65 (23.4)	0.047	200 (89.7)	242 (91.7)	244 (91.7)	243 (86.8)	0.470
Former	111 (48.9)	128 (46.6)	123 (46.2)	114 (41.0)		8 (3.6)	10 (3.8)	8 (3.0)	13 (4.6)	
Current	50 (22.0)	71 (25.8)	75 (28.2)	99 (35.6)		15 (6.7)	12 (4.6)	14 (5.3)	24 (8.6)	
**Education level (years, n, %)**										
≤ 9	30 (13.2)	34 (12.4)	41 (15.4)	41 (14.8)	0.494	40 (17.9)	48 (18.2)	51 (19.2)	62 (22.1)	0.971
10–15	125 (55.1)	162 (58.9)	135 (50.8)	161 (57.9)		167 (74.9)	194 (73.5)	194 (72.9)	200 (71.4)	
≥ 16	68 (30.0)	78 (28.4)	85 (32.0)	74 (26.6)		14 (6.3)	20 (7.6)	18 (6.8)	16 (5.7)	
Unknown	4 (1.8)	1 (0.4)	5 (1.9)	2 (0.7)		2 (0.9)	2 (0.8)	3 (1.1)	2 (0.7)	
**Body mass index (kg/m**^2^**, n, %)**										
< 18.5	8 (3.5)	7 (2.6)	5 (1.9)	6 (2.2)	0.776	16 (7.2)	18 (6.8)	15 (5.6)	20 (7.1)	0.110
18.5 to < 25.0	156 (68.7)	191 (69.5)	174 (65.4)	188 (67.6)		172 (77.1)	182 (68.9)	181 (68.1)	187 (66.8)	
≥ 25.0	63 (27.8)	77 (28.0)	87 (32.7)	84 (30.2)		35 (15.7)	64 (24.2)	70 (26.3)	73 (26.1)	
**Physical activity (METs・hours/day, mean ± SD)**	15.4 ± 15.4	15.7 ± 15.1	15.3 ± 15.4	13.0 ± 13.1	0.126	13.7 ± 12.7	13.1 ± 10.9	14.9 ± 12.9	14.5 ± 13.3	0.372
**Psychological stress (n, %)**										
Not at all	13 (5.7)	20 (7.3)	19 (7.1)	20 (7.2)	0.788	5 (2.2)	17 (6.4)	11 (4.1)	10 (3.6)	0.212
Not much	66 (29.1)	83 (30.2)	76 (28.6)	68 (24.5)		36 (16.1)	43 (16.3)	57 (21.4)	55 (19.6)	
A little	107 (47.1)	128 (46.6)	112 (42.1)	126 (45.3)		126 (56.5)	131 (49.6)	138 (51.9)	130 (46.4)	
A lot	40 (17.6)	43 (15.6)	57 (21.4)	62 (22.3)		55 (24.7)	73 (27.7)	59 (22.2)	84 (30.0)	
Unknown	1 (0.4)	1 (0.4)	2 (0.8)	2 (0.7)		1 (0.5)	0 (0.0)	1 (0.4)	1 (0.4)	
**Sleeping hours (hours/day, n, %)**										
< 6	15 (6.6)	24 (8.7)	23 (8.7)	34 (12.2)	0.072	28 (12.6)	33 (12.5)	31 (11.7)	40 (14.3)	0.569
6 to < 8	170 (74.9)	191 (69.5)	181 (68.1)	166 (59.7)		163 (73.1)	195 (73.9)	183 (68.8)	197 (70.4)	
≥ 8	42 (18.5)	60 (21.8)	61 (22.9)	77 (27.7)		32 (14.4)	36 (13.6)	52 (19.6)	42 (15.0)	
Unknown	0 (0.0)	0 (0.0)	1 (0.4)	1 (0.4)		0 (0.0)	0 (0.0)	0 (0.0)	1 (0.4)	
**Family history of hypertension (n, %)**										
Father	53 (23.4)	62 (22.6)	58 (21.8)	68 (24.5)	0.982	64 (28.7)	69 (26.1)	62 (23.3)	69 (24.6)	0.552
Mother	61 (26.9)	83 (30.2)	75 (28.2)	63 (22.7)	0.278	76 (34.1)	86 (32.6)	77 (29.0)	96 (34.3)	0.119
**Medical history (n, %)**										
Diabetes mellitus	21 (9.3)	29 (10.6)	26 (9.8)	17 (6.1)	0.460	10 (4.5)	10 (3.8)	12 (4.5)	11 (3.9)	0.247
Dyslipidemia	33 (14.5)	50 (18.2)	52 (19.6)	38 (13.7)	0.361	39 (17.5)	50 (18.9)	41 (15.4)	59 (21.1)	0.336
Cardiovascular disease	6 (2.6)	10 (3.6)	14 (5.3)	6 (2.2)	0.384	6 (2.7)	3 (1.1)	5 (1.9)	12 (4.3)	0.184
Stroke	4 (1.8)	10 (3.6)	9 (3.4)	9 (3.2)	0.733	5 (2.2)	3 (1.1)	4 (1.5)	12 (4.3)	0.013
**Plasma Hcy (nmol/mL, mean ± SD)**	6.4 ± 0.7	8.0 ± 0.4	9.6 ± 0.5	13.9 ± 5.2	< 0.001	5.0 ± 0.5	6.2 ± 0.3	7.3 ± 0.4	10.1 ± 2.9	< 0.001
**Plasma folate (ng/mL, mean ± SD)**	9.5 ± 2.9	8.9 ± 2.1	8.2 ± 2.0	7.5 ± 1.3	< 0.001	11.3 ± 4.4	9.7 ± 2.2	9.3 ± 3.0	8.6 ± 2.0	< 0.001
**Plasma vitamin B**_**12**_ **(pg/mL, mean ± SD)**	940 ± 345	868 ± 238	815 ± 235	754 ± 324	< 0.001	1070 ± 502	945 ± 357	899 ± 335	801 ± 209	< 0.001
**Dietary folate intake (μg/1000 kcal/day, mean ± SD)**	172 ± 51	169 ± 58	163 ± 48	156 ± 54	0.004	239 ± 87	236 ± 80	241 ± 81	228 ± 69	0.238
**Energy intake (kcal/day, mean ± SD)**	2000 ± 363	1949 ± 360	1937 ± 357	1901 ± 368	0.024	1566 ± 246	1576 ± 241	1531 ± 246	1513 ± 251	0.010
**Study area (n, %)**										
Okazaki	36 (15.9)	53 (19.3)	52 (19.6)	46 (16.6)	< 0.001	34 (15.3)	55 (20.8)	45 (16.9)	43 (15.4)	< 0.001
Shizuoka	102 (44.9)	101 (36.7)	74 (27.8)	61 (21.9)		45 (20.2)	42 (15.9)	30 (11.3)	17 (6.1)	
Takashima	37 (16.3)	38 (13.8)	26 (9.8)	26 (9.4)		81 (36.3)	69 (26.1)	62 (23.3)	58 (20.7)	
Kyoto	2 (0.9)	13 (4.7)	22 (8.3)	51 (18.4)		0 (0.0)	2 (0.8)	7 (2.6)	15 (5.4)	
Saga	40 (17.6)	42 (15.3)	36 (13.5)	44 (15.8)		54 (24.2)	60 (22.7)	59 (22.2)	47 (16.8)	
Kagoshima	10 (4.4)	28 (10.2)	56 (21.1)	50 (18.0)		9 (4.0)	36 (13.6)	63 (23.7)	100 (35.7)	

*Hcy* homocysteine, *METs* metabolic equivalents, *SD* standard deviation.

Table 2 presents the crude, age-adjusted and multivariate-adjusted ORs and 95% CIs for hypertension according to quartile levels of plasma Hcy, folate, and vitamin B_12_, and dietary folate intake. A total of 734 participants (404 men and 330 women, representing 35.3% of participants) had hypertension. Plasma Hcy level in those with hypertension was significantly higher than that in those without hypertension; the means ± SD (nmol/mL) were 8.8 ± 3.2 and 8.3 ± 3.6, respectively (*P* < 0.001). Plasma Hcy level was positively and significantly associated with the prevalence of hypertension, after controlling for multiple covariates including plasma levels of folate and vitamin B_12_, and dietary folate intake. The multivariate-adjusted ORs for the highest quartile group of plasma Hcy were 2.36 (95% CI 1.41–3.96) in men and 1.86 (95% CI 1.11–3.11) in women, as compared with the lowest group (*P* for trend = 0.014 and 0.005, respectively). Dietary folate intake was not correlated with hypertension in both men and women. The multivariate-adjusted OR for the highest quartile group of dietary folate intake was 0.65 (95% CI 0.41–1.05) in men and 0.93 (95% CI 0.58–1.51) in women, as compared with the lowest group (*P* for trend = 0.099 and 0.703, respectively). The multivariate-adjusted ORs for the second and highest quartile groups of plasma folate in men were significantly higher than unity; 2.22 (95% CI 1.40–3.51) and 1.98 (95% CI 1.23–3.21), respectively, as compared with the lowest group (*P* for trend = 0.231). The linear trend, however, was not statistically significant. The corresponding ORs were not significant in women. Plasma vitamin B_12_ level was positively associated with hypertension, especially in women. The multivariate-adjusted OR for the highest quartile group of plasma vitamin B_12_ was 1.49 (95% CI 0.93–2.37), as compared with the lowest group (*P* for trend = 0.027).

**Table 2:** The ORs and 95% CIs for hypertension according to quartile levels (Q1–Q4) of plasma Hcy, folate, and vitamin B_12_, and dietary folate intake, by sex.

	Men (n = 1046)					Women (n = 1033)				
	Q1	Q2	Q3	Q4	*P* for trend	Q1	Q2	Q3	Q4	*P* for trend
**Hcy**										
Median (nmol/mL)	6.6	8.0	9.4	12.4		5.2	6.2	7.4	9.3	
Range	3.4–7.2	7.4–8.6	8.8–10.6	10.8–59.0		3.0–5.6	5.8–6.6	6.8–8.0	8.2–40.4	
No. of participants	227	275	266	278		223	264	266	280	
No. of hypertension cases (n, %)	69 (30.4)	113 (41.1)	106 (39.9)	116 (41.7)		61 (27.4)	74 (28.0)	86 (32.3)	109 (38.9)	
Crude OR (95% CI)	1.00 (reference)	1.60 (1.10–2.32)	1.52 (1.04–2.21)	1.64 (1.13–2.37)	0.251	1.00 (reference)	1.03 (0.69–1.54)	1.27 (0.86–1.88)	1.69 (1.16–2.48)	0.006
Age-adjusted OR (95% CI)	1.00 (reference)	1.64 (1.11–2.42)	1.61 (1.09–2.39)	1.91 (1.29–2.83)	0.053	1.00 (reference)	1.04 (0.68–1.58)	1.21 (0.80–1.82)	1.59 (1.06–2.37)	0.012
Multivariate-adjusted OR^a^ (95% CI)	1.00 (reference)	1.71 (1.11–2.66)	1.56 (0.99–2.46)	2.01 (1.26–3.21)	0.046	1.00 (reference)	1.06 (0.67–1.69)	1.30 (0.82–2.06)	1.50 (0.93–2.42)	0.034
Multivariate-adjusted OR^b^ (95% CI)	1.00 (reference)	1.75 (1.12–2.75)	1.80 (1.12–2.90)	2.36 (1.41–3.96)	0.014	1.00 (reference)	1.17 (0.73–1.88)	1.48 (0.92–2.39)	1.86 (1.11–3.11)	0.005
**Folate**										
Median (ng/mL)	6.4	7.5	8.5	10.6		7.1	8.3	9.6	12.4	
Range	4.5–6.9	7.0–7.9	8.0–9.2	9.3–25.2		4.8–7.7	7.8–8.8	8.9–10.5	10.6–34.5	
No. of participants	244	255	268	279		257	248	267	261	
No. of hypertension cases (n, %)	70 (28.7)	107 (42.0)	106 (39.6)	121 (43.4)		73 (28.4)	75 (30.2)	91 (34.1)	91 (34.9)	
Crude OR (95% CI)	1.00 (reference)	1.80 (1.24–2.61)	1.63 (1.12–2.35)	1.90 (1.32–2.74)	0.118	1.00 (reference)	1.09 (0.75–1.60)	1.30 (0.90–1.89)	1.35 (0.93–1.96)	0.806
Age-adjusted OR (95% CI)	1.00 (reference)	2.03 (1.37–3.02)	1.57 (1.06–2.31)	1.62 (1.11–2.38)	0.688	1.00 (reference)	0.99 (0.66–1.48)	1.08 (0.73–1.60)	1.09 (0.74–1.62)	0.689
Multivariate-adjusted OR^a^ (95% CI)	1.00 (reference)	2.06 (1.32–3.22)	1.38 (0.88–2.15)	1.55 (0.99–2.41)	0.714	1.00 (reference)	1.08 (0.67–1.66)	1.14 (0.74–1.75)	1.16 (0.74–1.80)	0.884
Multivariate-adjusted OR^b^ (95% CI)	1.00 (reference)	2.22 (1.40–3.51)	1.58 (0.99–2.50)	1.98 (1.23–3.21)	0.231	1.00 (reference)	1.09 (0.69–1.73)	1.23 (0.79–1.91)	1.33 (0.83–2.11)	0.745
**Vitamin B**_**12**_										
Median (pg/mL)	620	735	855	1040		655	795	920	1170	
Range	400–685	690–785	790–925	930–4310		385–720	725–855	860–1000	1010–6040	
No. of participants	252	258	272	264		253	262	253	265	
No. of hypertension cases (n, %)	85 (33.7)	103 (39.9)	100 (36.8)	116 (43.9)		73 (28.9)	75 (28.6)	79 (31.2)	103 (38.9)	
Crude OR (95% CI)	1.00 (reference)	1.31 (0.91–1.87)	1.14 (0.80–1.64)	1.54 (1.08–2.20)	0.040	1.00 (reference)	0.99 (0.68–1.45)	1.12 (0.77–1.64)	1.57 (1.09–2.26)	0.001
Age-adjusted OR (95% CI)	1.00 (reference)	1.06 (0.72–1.56)	0.83 (0.57–1.22)	1.06 (0.72–1.55)	0.854	1.00 (reference)	0.95 (0.64–1.42)	0.97 (0.65–1.45)	1.19 (0.81–1.76)	0.116
Multivariate-adjusted OR^a^ (95% CI)	1.00 (reference)	1.06 (0.69–1.63)	0.91 (0.58–1.42)	1.08 (0.69–1.69)	0.421	1.00 (reference)	0.95 (0.61–1.49)	1.19 (0.75–1.88)	1.34 (0.85–2.09)	0.058
Multivariate-adjusted OR^b^ (95% CI)	1.00 (reference)	1.03 (0.66–1.60)	1.04 (0.66–1.65)	1.23 (0.77–1.96)	0.147	1.00 (reference)	0.97 (0.61–1.53)	1.26 (0.79–2.02)	1.49 (0.93–2.37)	0.027
**Dietary folate intake**										
Median (μg/1000 kcal/day)	112	143	174	222		160	205	245	311	
Range	54–128	128–158	158–192	192–547		72–186	186–226	226–267	267–960	
No. of participants	262	261	262	261		258	259	258	258	
No. of hypertension cases (n, %)	111 (42.4)	109 (41.8)	88 (33.6)	96 (36.8)		70 (27.1)	86 (33.2)	87 (33.7)	87 (33.7)	
Crude OR (95% CI)	1.00 (reference)	0.98 (0.69–1.38)	0.69 (0.48–0.98)	0.79 (0.56–1.13)	0.210	1.00 (reference)	1.34 (0.92–1.95)	1.37 (0.94–1.99)	1.37 (0.94–1.99)	0.077
Age-adjusted OR (95% CI)	1.00 (reference)	0.89 (0.61–1.29)	0.58 (0.39–0.84)	0.59 (0.40–0.86)	0.004	1.00 (reference)	1.14 (0.77–1.71)	1.00 (0.67–1.50)	0.93 (0.62–1.38)	0.775
Multivariate-adjusted OR^a^ (95% CI)	1.00 (reference)	0.84 (0.55–1.28)	0.60 (0.38–0.93)	0.65 (0.41–1.03)	0.057	1.00 (reference)	1.21 (0.77–1.90)	1.05 (0.67–1.66)	0.92 (0.57–1.47)	0.749
Multivariate-adjusted OR^b^ (95% CI)	1.00 (reference)	0.81 (0.53–1.25)	0.60 (0.38–0.94)	0.65 (0.41–1.05)	0.099	1.00 (reference)	1.21 (0.77–1.91)	1.04 (0.65–1.65)	0.93 (0.58–1.51)	0.703

*CI* confidence interval, *Hcy* homocysteine, *OR* odds ratio.

^a^Adjusted for age (continuous variable), smoking status, alcohol consumption, education level, body mass index, physical activity (continuous variable), psychological stress, sleeping hours, family history of hypertension in father, family history of hypertension in mother, medical history (diabetes mellitus, dyslipidemia, cardiovascular disease, and stroke), total energy intake (continuous variable), menstruation status (women only), and study area.

^b^Additionally adjusted for plasma levels of Hcy, folate, and vitamin B_12_, and dietary folate intake each other.

## Discussion

In the present study, we found that increased plasma Hcy level was significantly associated with the prevalence of hypertension in both men and women after controlling for multiple covariates including plasma levels of folate and vitamin B_12_, and dietary folate intake. Dietary folate intake was not associated with the prevalence of hypertension in both men and women.

Our finding for the association between Hcy level and hypertension is consistent with those from previous cross-sectional studies^12–15^. As a large previous cross-sectional study, the Third National Health and Nutrition Examination Survey in the United States demonstrated that those with the highest quintile group of plasma Hcy had a two- to three-fold OR of hypertension, in comparison with the lowest group^14^, which is similar to our findings. In men, the multivariate-adjusted OR of hypertension for the highest quintile group (range for plasma Hcy concentration: 13.1–132 μmol/L) was 1.9 (95% CI 0.71–5.14) in comparison with the lowest group (3.3–7.5 μmol/L) (*P* for trend = 0.12). In women, the OR for the highest quintile group (11.0–118 μmol/L) was 3.0 (95% CI 1.70–5.39) as compared with the lowest group (3.0–6.1 μmol/L) (*P* for trend = 0.0001).

There are several potential mechanisms by which increased plasma Hcy can cause hypertension. Hcy has been shown to be associated with oxidative stress, endothelial dysfunction, increased inflammation, and decreased bioavailability of endothelium-derived nitric oxide, resulting in increased blood pressure^9–11^. Additionally, Hcy activates metalloproteinase and induces collagen synthesis, and causes imbalances of elastin/collagen ratio. Interestingly, Hcy also promotes angiotensin-converting enzyme activity that may lead to upregulation of angiotensin II and subsequently hypertension^42^. Nevertheless, several cross-sectional and large prospective studies showed no evidence for an association between Hcy level and hypertension, after controlling for confounding factors^16–21^. The Mendelian randomization (MR) approach is a method of using the measured variation in genes to examine the causal effect of modifiable exposures for disease. The MR has recently been applied to evaluate this association. In that study, Hcy was reported not to be a causal factor for blood pressure^43^, suggesting that increased plasma Hcy may be concomitant with hypertension. However, the evidence from prospective studies and MR analysis is insufficient for this association; thus, further investigation will be needed to verify this relationship.

Although folate plays an important role in the metabolism of Hcy, limited data are available on the association between plasma folate levels and dietary folate intake and the risk of hypertension. In the present study, dietary folate intake was not associated with the prevalence of hypertension, whereas the ORs of hypertension were significantly higher than unity for the second and highest quartiles of plasma folate in men. The reason for this discrepancy is unclear. Cross-sectional and prospective studies have shown an inverse association between serum folate level and blood pressure^23,24^. In addition, two prospective studies have demonstrated that dietary folate intake is inversely associated with the incidence of hypertension^24,25^. Our findings are inconsistent with these results. When we calculated the Spearman correlation coefficient between plasma folate level and energy-adjusted dietary folate intake, the positive correlation appeared to be weak, which is similar to results from a previous study in Japan^44^. The biological mechanisms for the weak correlation between plasma folate level and dietary folate intake remained unclear. Measuring folate intake with a questionnaire and the plasma folate assay are prone to a different type and magnitude of systematic or random errors.

It is possible that unknown confounders brought significantly high ORs of hypertension for the second and highest quartiles of plasma folate level in men, although we controlled for multiple confounders to evaluate this association. As the trend was not significant in men and we observed no clear association of either dietary intake or plasma levels of folate with hypertension in women, further studies are warranted to examine the role of plasma folate.

We also found no clear association between plasma vitamin B_12_ levels and hypertension, although there was a positive correlation only in women. These results are consistent with those from a previous case–control study^45^. Interestingly, a cross-sectional study showed that dietary vitamin B_12_ intake is inversely associated with blood pressure among preschool children^26^, suggesting that vitamin B_12_ may play a role in reducing the risk of hypertension. However, we did not consider this issue owing to no available data for dietary vitamin B_12_ intake. Further studies using data for both dietary intake and plasma levels of vitamin B_12_ will elucidate the inconsistent associations with hypertension.

We found a significant positive association between Hcy level and the prevalence of hypertension, with consideration for folate and vitamin B_12_ as related to Hcy level. It is meaningful that the strong positive association remained even when controlling for dietary folate intake, and plasma levels of folate and vitamin B_12_. The association may be independent of folate and vitamin B_12_.

The strengths of the present study include its relatively large sample size, use of a validated FFQ for dietary folate and energy intake, and controlling for several confounders obtained from the self-administered questionnaire. Several potential limitations, however, should be mentioned. First, we were unable to determine the causal relationship between Hcy level and risk of hypertension because of the cross-sectional design of this study. Second, because most participants analyzed in the present study were Japanese, the generalizability of our findings to other populations remains to be explored. Third, although our FFQ was well validated using dietary record^33–36^, the validity correlation coefficient for dietary folate intake was not so high, so that the dietary folate intake is a rough estimate to rank the participants. Fourth, we did not consider the loss of dietary folate intake due to the cooking with heat for foods because of limited data. Fifth, we did not examine the associations with consideration for folate supplement use because no participant reported its use in the present analysis. Lastly, we could not consider the genetic background of the included individuals in relation to the association with risk of hypertension because it requires too much analysis to be included in one study. *Methylenetetrahydrofolate reductase* (*MTHFR*) C677T (rs1801133) is one of the important polymorphisms strongly associated with Hcy levels in the human body, whose T allele significantly reduces MTHFR activity, resulting in increased plasma Hcy levels. However, this variant itself has been reported not to be associated with blood pressure^43^. In addition, other genetic background regarding blood pressure appeared to be unrelated to Hcy levels in the present study because we observed no clear differences in family history of parental hypertension among the quartiles of plasma Hcy level, by sex.

In conclusion, we found a significant positive association between plasma Hcy levels and the prevalence of hypertension when considering plasma folate and vitamin B_12_, and dietary folate intake typically involved in the metabolism of Hcy. Further studies are warranted to explore the causal inference for this association and to clarify the roles of folate and vitamin B_12_ in the risk of hypertension as related to Hcy.

## Data Availability

The datasets generated during and/or analyzed during the current study are not publicly available due to ethical restriction but are available from the corresponding author on reasonable request.

## References

[1] Kearney PM (2005). Global burden of hypertension: analysis of worldwide data. Lancet.

[2] Xi B (2012). Physical activity modifies the associations between genetic variants and hypertension in the Chinese children. Atherosclerosis.

[3] Hall ME (2014). Obesity, hypertension, and chronic kidney disease. Int. J. Nephrol. Renovasc. Dis..

[4] Shimbo D (2013). The contributions of unhealthy lifestyle factors to apparent resistant hypertension: findings from the reasons for geographic and racial differences in stroke (regards) study. J. Hypertens..

[5] Batis C (2013). Sodium intake from various time frames and incident hypertension among Chinese adults. Epidemiology.

[6] O'Donovan C (2014). Inverse relationship between physical activity and arterial stiffness in adults with hypertension. J. Phys. Act Health.

[7] Ganguly P, Alam SF (2015). Role of homocysteine in the development of cardiovascular disease. Nutr. J..

[8] Li J (2015). H-type hypertension and risk of stroke in Chinese adults: a prospective, nested case-control study. J. Transl. Int. Med..

[9] Rodrigo R, Passalacqua W, Araya J, Orellana M, Rivera G (2003). Homocysteine and essential hypertension. J. Clin. Pharm..

[10] Sutton-Tyrrell K, Bostom A, Selhub J, Zeigler-Johnson C (1997). High homocysteine levels are independently related to isolated systolic hypertension in older adults. Circulation.

[11] Tyagi N, Moshal KS, Lominadze D, Ovechkin AV, Tyagi SC (2005). Homocysteine-dependent cardiac remodeling and endothelial-myocyte coupling in a 2 kidney, 1 clip Goldblatt hypertension mouse model. Can. J. Physiol. Pharmacol..

[12] Refsum H (2006). The Hordaland Homocysteine Study: a community-based study of homocysteine, its determinants, and associations with disease. J. Nutr..

[13] Li WX (2017). Interactions of methylenetetrahydrofolate reductase gene polymorphisms, folate, and homocysteine on blood pressure in a Chinese hypertensive population. Clin. Lab..

[14] Lim U, Cassano PA (2002). Homocysteine and blood pressure in the Third National Health and Nutrition Examination Survey, 1988–1994. Am. J. Epidemiol..

[15] Li Z (2016). Hyperhomocysteinemia independently associated with the risk of hypertension: a cross-sectional study from rural China. J. Hum. Hypertens..

[16] Fakhrzadeh H (2005). Plasma homocysteine concentration and blood pressure in healthy Iranian adults: the Tehran Homocysteine Survey (2003–2004). J. Hum. Hypertens..

[17] Dinavahi R, Cossrow N, Kushner H, Falkner B (2003). Plasma homocysteine concentration and blood pressure in young adult African Americans. Am. J. Hypertens..

[18] Sundström J (2003). Plasma homocysteine, hypertension incidence, and blood pressure tracking: the Framingham Heart Study. Hypertension.

[19] Bowman TS, Gaziano JM, Stampfer MJ, Sesso HD (2006). Homocysteine and risk of developing hypertension in men. J. Hum. Hypertens..

[20] Forman JP, Choi H, Curhan GC (2009). Uric acid and insulin sensitivity and risk of incident hypertension. Arch. Intern. Med..

[21] Wang Y (2014). Homocysteine as a risk factor for hypertension: a 2-year follow-up study. PLoS ONE.

[22] Wang WW, Wang XS, Zhang ZR, He JC, Xie CL (2017). A Meta-analysis of folic acid in combination with anti-hypertension drugs in patients with hypertension and hyperhomocysteinemia. Front. Pharmacol..

[23] Shen M (2016). Serum folate shows an inverse association with blood pressure in a cohort of Chinese women of childbearing age: a cross-sectional study. PLoS ONE.

[24] Xun P (2012). Folate intake and incidence of hypertension among American young adults: a 20-y follow-up study. Am. J. Clin. Nutr..

[25] Forman JP, Rimm EB, Stampfer MJ, Curhan GC (2005). Folate intake and the risk of incident hypertension among US women. JAMA.

[26] Tamai Y (2011). Dietary intake of vitamin B_12_ and folic acid is associated with lower blood pressure in Japanese preschool children. Am. J. Hypertens..

[27] Stover PJ (2004). Physiology of folate and vitamin B_12_ in health and disease. Nutr. Rev..

[28] Hamajima N, J-MICC Study Group (2007). The Japan multi-institutional collaborative cohort study (J-MICC Study) to detect gene-environment interactions for cancer. Asian Pac. J. Cancer Prev..

[29] Wakai K (2011). Profile of participants and genotype distributions of 108 polymorphisms in a cross-sectional study of associations of genotypes with lifestyle and clinical factors: a project in the Japan multi-institutional collaborative cohort (J-MICC) study. J. Epidemiol..

[30] Higashibata T (2012). eNOS genotype modifies the effect of leisure-time physical activity on serum triglyceride levels in a Japanese population. Lipids Health Dis..

[31] Tamura T (2015). No association between *Helicobacter**pylori* infection and diabetes mellitus among a general Japanese population: a cross-sectional study. Springerplus.

[32] Sasakabe T (2018). Modification of the associations of alcohol intake with serum low-density lipoprotein cholesterol and triglycerides by ALDH2 and ADH1B polymorphisms in Japanese men. J. Epidemiol..

[33] Tokudome S (2004). Development of a data-based short food frequency questionnaire for assessing nutrient intake by middle-aged Japanese. Asian Pac. J. Cancer Prev..

[34] Tokudome Y (2005). Relative validity of a short food frequency questionnaire for assessing nutrient intake versus three-day weighed diet records in middle-aged Japanese. J. Epidemiol..

[35] Goto C (2006). Validation study of fatty acid consumption assessed with a short food frequency questionnaire against plasma concentration in middle-aged Japanese people. Scand. J. Nutr..

[36] Imaeda N (2007). Reproducibility of a short food frequency questionnaire for Japanese general population. J. Epidemiol..

[37] Brown CC (1994). Energy adjustment methods for nutritional epidemiology: the effect of categorization. Am. J. Epidemiol..

[38] Tamura T (2018). Association of exposure level to passive smoking with hypertension among lifetime nonsmokers in Japan: a cross-sectional study. Medicine (Baltimore).

[39] Nygård O (1995). Total plasma homocysteine and cardiovascular risk profile. The Hordaland Homocysteine Study. JAMA.

[40] Shimamoto K (2014). The Japanese society of hypertension guidelines for the management of hypertension (JSH 2014). Hypertens. Res..

[41] Black HR (2015). Eligibility and disqualification recommendations for competitive athletes with cardiovascular abnormalities: task force 6: hypertension: a scientific statement from the American heart association and the American college of cardiology. Circulation.

[42] Sen U, Mishra PK, Tyagi N, Tyagi SC (2010). Homocysteine to hydrogen sulfide or hypertension. Cell Biochem. Biophys..

[43] Borges MC, Hartwig FP, Oliveira IO, Horta BL (2016). Is there a causal role for homocysteine concentration in blood pressure? A Mendelian randomization study. Am. J. Clin. Nutr..

[44] Iso H (2003). Validity of the self-administered food frequency questionnaire used in the 5-year follow-up survey for the JPHC Study to assess folate, vitamin B6 and B_12_ intake: comparison with dietary records and blood level. J. Epidemiol..

[45] Scazzone C (2014). Correlation between low folate levels and hyperhomocysteinemia, but not with vitamin B_12_ in hypertensive patients. Ann. Clin. Lab. Sci..

